# A Comparison of Differential Gene Expression in Response to the Onset of Water Stress Between Three Hybrid Brachiaria Genotypes

**DOI:** 10.3389/fpls.2021.637956

**Published:** 2021-03-19

**Authors:** Charlotte Jones, Jose De Vega, Margaret Worthington, Ann Thomas, Dagmara Gasior, John Harper, John Doonan, Yuan Fu, Maurice Bosch, Fiona Corke, Jacobo Arango, Juan Andres Cardoso, Juan de la Cruz Jimenez, Ian Armstead, Narcis Fernandez-Fuentes

**Affiliations:** ^1^Institute of Biological, Environmental and Rural Sciences, Aberystwyth University, Aberystwyth, United Kingdom; ^2^Earlham Institute, Norwich, United Kingdom; ^3^Department of Horticulture, University of Arkansas, Fayetteville, AR, United States; ^4^International Center for Tropical Agriculture, Cali, Colombia; ^5^Tropical Forage Program, Alliance Biodiversity-CIAT, Cali, Colombia; ^6^School of Agriculture and Environment, Faculty of Science, The University of Western Australia, Crawley, WA, Australia

**Keywords:** *Brachiaria*, drought, differentially expressed genes (DEGs), comparative transcriptomics, functional enrichment

## Abstract

*Brachiaria* (Trin.) Griseb. (syn. *Urochloa* P. Beauv.) is a C_4_ grass genus belonging to the Panicoideae. Native to Africa, these grasses are now widely grown as forages in tropical areas worldwide and are the subject of intensive breeding, particularly in South America. Tolerance to abiotic stresses such as aluminum and drought are major breeding objectives. In this study, we present the transcriptomic profiling of leaves and roots of three *Brachiaria* interspecific hybrid genotypes with the onset of water stress, Br12/3659-17 (gt-17), Br12/2360-9 (gt-9), and Br12/3868-18 (gt-18), previously characterized as having good, intermediate and poor tolerance to drought, respectively, in germplasm evaluation programs. RNA was extracted from leaf and root tissue of plants at estimated growing medium water contents (EWC) of 35, 15, and 5%. Differentially expressed genes (DEGs) were compared between different EWCs, 35/15, 15/5, and 35/5 using DESeq2. Overall, the proportions of DEGs enriched in all three genotypes varied in a genotype-dependent manner in relation to EWC comparison, with intermediate and sensitive gt-9 and gt-18 being more similar to each other than to drought tolerant gt-17. More specifically, GO terms relating to carbohydrate and cell wall metabolism in the leaves were enriched by up-regulated DEGs in gt-9 and gt-18, but by down-regulated DEGs in gt-17. Across all genotypes, analysis of DEG enzyme activities indicated an excess of down-regulated putative apoplastic peroxidases in the roots as water stress increased. This suggests that changes in root cell-wall architecture may be an important component of the response to water stress in *Brachiaria.*

## Introduction

For healthy development, growth and reproduction plants need sufficient water. However, due to their sessile nature, plants often encounter unfavorable environmental conditions during their life cycles and water stress is a major environmental factor that limits crop growth and yield. Around one third of the planet is arid to semi-arid, with periodic drought affecting most of the rest of the landmass. As climate changes, more areas are being affected by water stress and for longer periods, and this poses major challenges for global agriculture. Therefore, understanding whole plant and molecular mechanisms that influence responses to water stress is of significant interest to plant scientists and breeders in seeking to maintain and improve crop yields.

*Brachiaria* (Trin.) Griseb. (syn. *Urochloa* P.Beauv*.)* is a C_4_ grass genus belonging to the Panicoideae (Renvoize et al., 1996). This genus includes several species which are important as agricultural grasses, notably *B. decumbens* Stapf., *B. brizantha* (Hochst. ex A. Rich.), *B. humidicola* (Rendle) Schweick and *B. ruziziensis* (R. Germ. and C. M. Evrard). These grasses, native to Africa, are now widely grown in the form of individual species and hybrids, as forage grasses in tropical areas worldwide (Keller-Grein et al., 1996). A number of *Brachiaria* species (referred to collectively as Brachiaria from this point forward) have been the subject of intensive breeding efforts. It is estimated that resultant forage varieties, many of them developed at the International Centre for Tropical Agriculture (CIAT), Colombia, now cover an area of 25 million hectares of agricultural land in Latin America^1^ and a further 99 million hectares in Brazil (Jank et al., 2014). In addition, opportunities for expanding the use of Brachiaria in Africa and Asia are currently being explored (Maass et al., 2015; Bui Van and Ba, 2019). Particular aims of breeding for these grasses have been to maintain and improve forage quality while increasing tolerances to abiotic stresses such as aluminum (acid soils) and drought, in addition to disease and pest resistance.

Much of the published research on the agriculturally important Brachiaria cultivars has focused either on agronomy and physiological evaluations of trait performance and response to abiotic and biotic stresses and exploring the genetics and cell biology of apomixis. In terms of abiotic stress, one of the reasons for the widespread use of Brachiaria cultivars as forage grasses is that they are often considered to be able to maintain productivity and ground cover under water-limited conditions (Petter et al., 2013; Pizarro et al., 2013; Cheruiyot et al., 2018) and a number of studies have recorded physiological responses of Brachiaria genotypes in response to drought. Observations include that hybrid Brachiaria cultivar Mulato II (*B. ruziziensis* x *B. brizantha*) manifests a ‘water saving’ strategy under imposed water-limitation in comparison to Napier grass (*Pennisetum purpureum*). This strategy includes closure of stomata, leaf rolling and reduced transpiration rates at relatively high soil moisture contents (Cardoso et al., 2015). In addition to these physiological responses, in a comparison of the *B. brizantha* cultivars Marandu and BRS Piatã, increased production of roots at lower soil levels and increased leaf senescence were also observed in response to water stress (Santos et al., 2013). A further study comparing five different Brachiaria species (*B. brizantha*, *B. decumbens*, *B. mutica*, *B. humidicola*, and *B. dictyoneura*) has indicated that differences in overall growth rates, root distributions, osmotic adjustments and timings of stomatal closures could all contribute to variations in drought tolerances (Guenni et al., 2002, 2004). Thus, it is established that there exists appreciable physiological variation in response to water-limitation within Brachiaria, which can be exploited in improving drought tolerance.

Despite the widespread importance of Brachiaria in tropical agriculture, it can still be considered an ‘orphan crop’ in terms of the resources for molecular genetics, biological and genomic analyses. Much of the published work has either focused on identifying the apomixis locus and linked markers which may be useful in plant breeding (Tohme et al., 1996; Pessino et al., 1997, 1998; Zorzatto et al., 2010; Thaikua et al., 2016; Worthington et al., 2016; Worthington et al., 2019) or generating improved understanding of the molecular phylogeny of the species group (recent references include Pessoa-Filho et al., 2015, 2017; Triviño et al., 2017; Kuwi et al., 2018; Tegegn et al., 2019). Again, because of the modest resources available for research on tropical forages, only recently have comprehensive RNAseq-based gene expression studies for Brachiaria been published. These consist of comparative leaf transcriptomics of two highly divergent *B. humidicola* genotypes (Vigna et al., 2016) and differential gene expression in a *B. decumbens* genotype exposed to aluminum (Salgado et al., 2017). However, recently, a major landmark in Brachiaria research has been the release of the first draft genome, derived from a diploid *B. ruziziensis* accession. This release was accompanied by a comprehensive gene annotation and a study of differential gene expression in response to aluminum in both *B. ruziziensis* and *B. decumbens* (Worthington et al., 2020).

While many studies on differential gene expression in response to water stress have been published on grass species, particularly the major cereals, there is only a limited body of knowledge on water stress-related changes in patterns of gene expression in forage grasses (Foito et al., 2009; Liu and Jiang, 2010; Pan et al., 2016; Zhao et al., 2016; Zhu et al., 2017; Ji et al., 2018; Dinkins et al., 2019; Fradera-Sola et al., 2019), the majority of which focus on temperate C_3_ forages. Drought tolerance of breeding selections is routinely evaluated in the CIAT forage breeding program, including measuring water extraction under progressive drying soil conditions – for which variation exists across apomictic Brachiaria germplasm. As described previously, the orphan status of Brachiaria in terms of molecular genetic evaluation means that little is known about gene expression responses linked to the onset of water stress in this genus. Our motivation for the present study was to begin to address this lack of information. Thus, using an experimental system which allowed for progressive sampling of both leaves and roots in a drying growing medium, we have undertaken a bioinformatic comparison of patterns of differential gene expression between three Brachiaria genotypes with different tolerance to water stress in CIAT evaluations, in order to examine the nature and conservation of these responses.

## Materials and Methods

### Plant Material and Growth Conditions

Three Brachiaria hybrid breeding selections developed at CIAT, which have shown contrasting responses to an imposed drought condition, were used in this study (Supplementary Table 1). These included Br12/3659-17 (gt-17), Br12/2360-9 (gt-9), and Br12/3868-18 (gt-18) previously characterized as having good, intermediate and poor tolerance to drought, respectively, in evaluations conducted in the CIAT breeding program. Plants and seeds used in this study were obtained directly from the CIAT tropical forage breeding program.

The three hybrids used were developed from an interspecific recurrent selection program focused on developing improved apomictic Brachiaria cultivars by crossing a synthetic, fully sexual breeding population with a non-inbred apomictic tester (Miles, 2007). The recurrent selection population was developed by crossing a sexually reproducing synthetic autotetraploid accession of *B. ruziziensis* with nine apomictic tetraploid accessions of *B. decumbens* and *B. brizantha* and recombining their sexual progeny during nine cycles of open pollination between 1992 and 2011. All the wild accessions used to initiate the CIAT Brachiaria breeding program are publicly available in the CIAT Genebank^2^. The three breeding selections chosen for this experiment are apomictic progeny of sexual selections from the ninth cycle of recurrent selection with the apomictic tester *B. decumbens* CIAT 606 (cv. Basilisk).

One seed per accession was sown in John Innes potting compost and germinated and grown in a climate-controlled cabinet under a photoperiod of 12 h per day with 60% relative humidity at 25°C constant temperatures. When the plants were large enough, they were split and left to grow in the control cabinet. After establishment, the four most similar looking clones from each accession were split again into three further clones, and nine clones per accession were chosen for the experiment (three sampling points and three replicates per sampling point). The roots of the nine clones were cleaned of soil, placed in vermiculite, and watered with a nutrient solution consisting of: [μM], NH_4_NO_3_ [500], KNO_3_ [300], Ca[NO_3_] [200], NaH_2_PO_4_ [5], MgCl_2_ [90], MgSO_4_ [60], FeCl_3_ [5], Na-EDTA [5], H_3_B0_3_ [6], MnSO_4_ [1], ZnSO_4_ [1], CuSO_4_ [0.2], Na_2_MoO_4_ [1], Na_2_SiO_3_ [5], NaCl [55], adjusted to pH 4.2 using 1M HCl. This nutrient solution, mimicking the pH of acid soils, was developed at CIAT to encourage root growth in Brachiaria (based on Wenzl et al., 2003, 2006). When the plants had recovered from transplantation, they were watered to full capacity and then, with no further watering, the fall in estimated growing medium water content (EWC) was measured using a HH2 Delta-T meter (AT Delta-T devices, Cambridge, United Kingdom). Leaf and root material were sampled at 35% (full capacity), 15% and 5% EWC (c. 15 days). Leaves were sampled directly into liquid nitrogen and root material was washed briefly in distilled water to remove vermiculite and then placed in liquid nitrogen and stored at –80°C prior to extraction of RNA.

### Leaf Relative Water Content

Estimations of leaf relative water content (RWC) were measured at 35, 15, 5, and 1% EWC (RNA extraction was not carried out at 1%). RWC estimations were carried out on additional replicates of the same genotypes prepared and grown identically to the plants used for RNA extraction, as follows. Three leaves from each replicate were removed, an 8 cm mid-section was cut from each leaf, and the fresh weight (FW) was measured. This excised section was then placed in a 50 ml tube containing 5 ml water, capped and left at 4°C for 24 h. After this period, the leaf sections were blotted and turgid weight (TW) was measured. The sections were then dried for 24 h at 80°C for the dry weight (DW). RWC was calculated as (FW-DW/TW-DW) × 100.

### RNA Extraction and cDNA Library Construction and Sequencing

RNA extraction, library construction and sequencing was carried out on each replicate independently (i.e., tissues and samples were not pooled). RNA was extracted from the root and leaf material, using a hot phenol technique (Ougham and Davies, 1990), and suspended in 100 μl of sterile 0.5M TE buffer. Quantification of the RNA samples was performed on a NanoDrop^®^ 1000 (Thermo, Waltham, MA, United States) at a wavelength of 260 nm. RNA sample quality was evaluated using the A_260 *nm*_/A_280 *nm*_ wavelength ratio and by direct observation on a 1% agarose gel. Sequencing libraries were constructed and sequenced at the DNA sequencing Center, Brigham Young University, Provo, UT, United States using Illumina kits for either Poly-A selected or Ribo-Zero rRNA removal. Illumina sequencing was performed using a HiSeq^TM^ 2000 platform according to the manufacturer’s instructions (Illumina, San Diego, CA, United States).

### RNAseq Processing and Quality Control and Mapping

Prior to mapping, raw reads were processed using Trimmomatic v.0.33 (Bolger et al., 2014) to remove adapters using the following parameters (optimized after several run tests): ILLUMINACLIP:TruSeq3-PE-2.fa LEADING:15 SLIDINGWINDOW:4:15 MINLEN:30 HEADCROP:12, and the quality of resulting trimmed and cleaned reads was assessed using FastQC v.0.11 (Wingett and Andrews, 2018). Reads were then mapped to the assembly version of the Brachiaria genome (Worthington et al., 2020) using the splice-aware mapper Hitsat2 v.2.0.0 (Kim et al., 2015).

### Pre-Processing and Quantification of Transcripts

Prior to calling of differentially expressed genes (DEGs) a pre-processing filtering was performed to remove potential artifacts and assess the quality of the replicates. Count matrices were derived from bam files above using the *GenomicFeatures* and *GenomicAlignments* R libraries. Transcripts with a count lower than one in any sample were discarded. We applied the regularized logarithm transformation (rlog) as implemented in the DESeq2 package to decrease the variance among gene expression values (Love et al., 2014) and then calculated a distance matrix between samples and performed a principle component analysis (PCA) to quantify experimental covariates and batch effects among samples and replicates (Fisher et al., 2004).

### Estimating the Completeness of Transcriptomes

The transcriptome in each sample was assessed for its completeness as a measure of quality of the sequencing. Clean reads were mapped to the reference genome and assembled using StringTie v1.1.0 (Pertea et al., 2015) using default parameters. The completeness of each transcriptome was assessed using BUSCO (Simao et al., 2015) on the *early_release plantdb* set, composed of 1440 core genes.

### Identification of Differentially Expressed Genes

Quantification of transcripts was done using Salmon (Patro et al., 2017) using precomputed mapping files (bam files) generated as described above using the *–ValidateMappings –gcBias* and –numBootstraps set to 1000 to improve the quantification. Derived counts were used as inputs to call DEGs using DESeq2 across three EWC-point comparisons: 35% vs. 15% (35/15), 35% vs. 5% (35/5) and 15% vs. 5% (15/5) for the three genotypes independently both in shoot and root tissues. Genes with a log_2_ fold change (LFC) above one and a false discovery rate (FDR) of ≤5% were considered as DEGs.

Differentially expressed genes were categorized according to pattern of expression and up- or down-regulation over the three sampling point comparisons and assigned an expression category. Each expression category was defined by a three-letter code: the first letter indicates whether the DEGs contributing to the enrichment of the indicated GO terms were up- (u) or down- (d) regulated at comparison point 35/15, the second letter at 35/5 and the third letter at 15/5. The letter n at position one, two or three indicates that the GO term was not significantly enriched at that comparison point. E.g., GO terms in expression category *d-d-n* were associated with down-regulated DEGs at 35/15 and 35/5 but were not-significantly enriched at 15/5 by either up- or down-regulated DEGs; GO terms in expression category *n-n-u* were associated with up-regulated DEGs at 15/5 but were not-significantly enriched at either 35/15 or 35/5.

### Functional Annotation of Differentially Expressed Genes

The reference genome was functionally re-annotated using Blast2GO 5.25 (Pro) (Conesa and Gotz, 2008) as a prior step before computing Gene Ontology (GO) term enrichments. The functional annotation was done as follows: BLAST searches were performed on the nr database (release March 2019) using BLASTx command from ncbi-blast-2.2.28 + release (Camacho et al., 2009) at an *E*-value cut-off of 1 × 10^–6^ and selecting the top 20 hits. InterPro searches were performed using InterProScan v.5.18-57 (Jones et al., 2014) on TIGRFAM (Haft et al., 2013), PFAM (Finn et al., 2016), SMART (Letunic et al., 2006), PANTHER (Mi et al., 2013), and Gene3d (Lees et al., 2010) databases.

### Identification of GO Terms and Mapping of Enzyme Codes to KEGG Pathways

Gene Ontology term enrichment was calculated for each set of DEGs associated with each three letter expression category (see section “Identification of differentially expressed genes”) for each genotype, using Blast2GO 5.25 (Pro) (Conesa and Gotz, 2008) and a 5% FDR cut-off threshold. GO terms were subsequently grouped into putative functional hierarchies visualized at https://www.ebi.ac.uk/QuickGO/slimming.

The complete lists of GO terms were further filtered according to either of two criteria in order to focus on the best-supported enrichments: (1) GO terms which were associated with differential gene expression in all three genotypes, or (2) GO terms that were within the top 10% of most significantly enriched GO terms within the original 5% FDR (Fisher’s exact test *p*-value cut-off thresholds of –log_10_ *p* = 10.4 and *p* = 8 for leaf and root, respectively).

Mapping of enzyme codes to KEGG pathways (Kanehisa and Goto, 2000) was accomplished using the relevant module contained within Blast2GO.

## Results

### Relative Water Content

Supplementary Figure 1 illustrates the change in RWC for the three hybrid Brachiaria genotypes as the EWC of the medium decreased. Major changes in RWC for all 3 genotypes only occurred after the 5% EWC point was reached. No significant changes in RWC were observed with decreasing EWC between 35 and 5% for gt-9 and gt-18, however, gt-17 did show a significant decrease in RWC over the same range (*P* < 0.01). These results indicated the onset of water-stress for the genotypes at around 5% EWC.

### Pre-processing, Mapping, and Quality of Sequencing and Replicates

RNA libraries were processed and sequenced in a single batch yielding an average of c. 11M reads per replicate for both leaf and root tissue, with a maximum and minimum of 13.2 and 10.1M reads for any individual replicate. The drop-off rate upon trimming and quality control (discarding low quality and non-paired reads) was less than 1%. The mapping rate of the retained reads to the reference genome (Worthington et al., 2020) varied between 76 and 58% for leaf tissue and 72 and 44% for the root tissue (Supplementary Table 2). From the BUSCO analysis, the completeness of the transcriptome across all samples was estimated at an average of c. 77% in terms of complete and partial gene coverage. This compares with 85% for the Brachiaria reference genome (Worthington et al., 2020) (Supplementary Table 3).

The variability of gene expression based on normalized counts among the replicates is illustrated in the form of density and principal component analysis (PCA) plots (Supplementary Figures 2, 3). The density plots of the rlog transformation of normalized counts both in leaf and root samples were very homogenous and, thus, indicate little variability among replicates (Supplementary Figures 2A–F). This is also reflected in the PCA plots (Supplementary Figure 3), which show that within each EWC category the different replicates tended to cluster together, with the first two principle components accounting for 62–87% of the total variability. The exception to this was for gt17, which showed less tight clustering of the replicates at 15% EWC (leaf; Supplementary Figure 3B) and 35% EWC (root; Supplementary Figure 3E). In terms of the first principle component, the analysis showed that the distance between samples was greatest for the 35 and 5% EWC sampling points and that the 15% sampling point was intermediate between 35 and 5%. It would appear, therefore, that the change in EWC was the main driver explaining the overall variability among samples.

### Distribution of Differentially Expressed Genes Over Genotypes and Comparison Points

Leaf and root transcriptomes were analyzed for the presence of DEGs across three EWC-point comparisons, 35/15, 35/5, and 15/5 for the three genotypes independently. The total numbers of up- and down-regulated DEGs at any stage and at each of the comparison points is given in Table 1. On average across genotypes at any time, 2,898 and 2,595 DEGs were identified in leaves and root, respectively, evenly distributed between up- and down-regulated DEGs. The total number of DEGs associated with the individual genotypes was quite variable with c. twice as many DEGs identified in the leaf tissue for gt-18 as compared to gt-9 and c. three-times as many DEGs identified in gt-9 as compared to gt-17 in the root tissue. For the comparison points, the highest numbers of DEGs were associated with the 35/5 comparison in both leaves and roots; and the smallest number with the 35/15 comparison in leaves and the 15/5 comparison in roots. Out of the total of 35,196 gene models included in the analysis, 5,821 were significantly differentially up- or down-regulated in any of the genotypes at any of the comparison points in the leaf tissue and 5,322 in the root tissue, with 2,118 specific gene models being present as DEGs in both leaves and roots (Supplementary Figure 4). The relative distributions of DEGs between and among the three genotypes in leaves and roots is described in Supplementary Figure 5 and the complete lists of DEGs associated with genotypes and comparison points for both leaf and root is given in Supplementary File 1.

**TABLE 1:** The total number of up- and down-regulated differentially expressed genes at all comparison points and at the individual comparison points for each genotype and tissue.

	Genotype	Overall comparison points				Individual comparison point^1^								
						35/15			35/5			15/5		
			
		Up	Down	Up down	Total	Up	Down	Total	Up	Down	Total	Up	Down	Total
**Leaf**	**gt-9**	905	883	66	1854	216	371	587	720	774	1494	408	169	577
	**gt-17**	1340	1379	4	2723	67	20	87	696	1120	1816	937	923	1860
	**gt-18**	2157	1915	44	4116	363	161	524	1980	1289	3269	937	591	1528
	**mean**	1467	1392	38	2898	215	184	399	1132	1061	2193	761	561	1322
**Root**	**gt-9**	1645	1957	247	3849	819	1413	2232	1074	1434	2508	746	359	1105
	**gt-17**	578	666	1	1245	69	127	196	555	636	1191	109	56	165
	**gt-18**	1018	1536	137	2691	279	791	1070	695	1014	1709	716	504	1220
	**mean**	1080	1386	128	2595	389	777	1166	775	1028	1803	524	306	830

*^1^35/15, 35/5, and 15/5 indicate differential gene expression in leaves or roots comparing transcriptomes at 35 and 15%, 35 and 5%, and 15 and 5% estimated growing medium water contents, respectively.*

**FIGURE 1:**
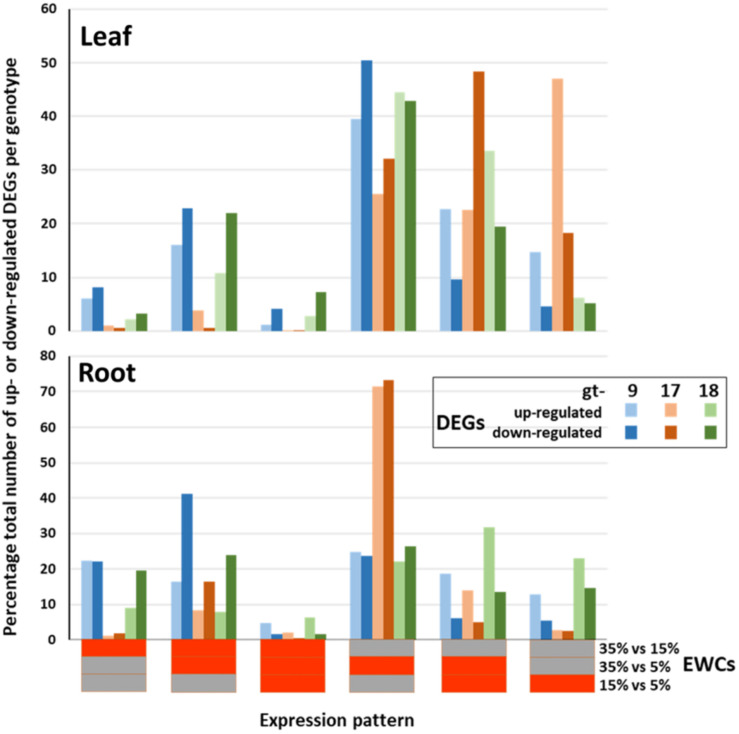
Relative proportions of differentially expressed genes (DEGs) across genotypes (gt-) 9, 17 and 18 and combined estimated water content (EWC) comparison points (expression patterns). The percentage proportions of up- and down-regulated DEGs identified for each individual expression pattern are indicated by the colored columns. Horizontal bars beneath the *x* axis indicate the individual EWC comparison points (indicated at the end of the *x* axis) in which the genes were differentially expressed for each expression pattern; red, differentially expressed for that EWC comparison point; gray, not differentially expressed for that EWC comparison point. E.g., the first expression pattern indicates the percentage proportion of the total number of up- and down-regulated DEGs for each genotype that were differentially expressed for the 35% vs. 15% comparison point (red) but were not differentially expressed for the 35% vs. 5% and 15% vs. 5% comparison points (gray).

The overall patterns of differential gene expression varied between genotypes and between leaves and roots (Figure 1). In the leaves, while all the genotypes were similar in having high proportions of their DEGs present in just the 35/5 comparison stage, differences between the genotypes were also apparent. For example, for gt-9 and gt-18 between c. 10 and 22% of their up- and down-regulated DEGs were detected in both the 35/15 and 35/5 comparison stages, whereas the equivalent figures for gt-17 were < 5%. Also, almost 50% of the DEGs for gt-17 were identified only in the 15/5 comparison stage; for gt-9 and gt-18 the equivalent figures were <15% and <5%, respectively. For roots, gt-9 and gt-18 showed more even distributions of DEGs across all of the comparison stages. However, this was in contrast to gt-17, in which 72% of the DEGs were present only in the 35/5 comparison stage. In summary, patterns of differential gene expression were similar in gt-9 and gt-18 and this differed from the pattern seen in gt-17. Overall, for all genotypes, the patterns of differential expression were different between leaves and roots.

### Association of DEGs With Enriched GO Terms

The DEGs identified within each category were analyzed for association with GO terms on an individual genotype basis using a 5% FDR. Across the three genotypes, a total of 1,210 significantly enriched GO terms (referred to as GO terms from this point forward) were identified from the leaf DEGs and 856 from the root DEGs. For the individual genotypes the total numbers of GO terms were 280, 617, and 722 for leaves and 420, 212 and 467 for roots for gt-9, gt-17 and gt-18, respectively (a complete lists of GO terms for individual genotypes for leaf and root are provided in Supplementary Files 2, 3 respectively). Of the total numbers of GO terms associated with leaf tissue, 137 and 85 were enriched for all three genotypes in leaves and roots, respectively. The number of DEGs identified for each comparison stage, the number of associated enriched GO terms and the percentage proportion of the DEGs which contribute to these enriched GO terms is summarized in Table 2.

**TABLE 2:** Summary descriptions of the numbers and proportions of differentially expressed genes (DEGS) according to genotype (gt) and expression category.

		Expression category^1^	gt-9				gt-17				gt-18			
			GOs^2^	DEGs^3^	% DEGs^4^	Total DEGs^5^	GOs	DEGs	% DEGs	Total DEGs	GOs	DEGs	% DEGs	Total DEGs
**Leaf**	**down- regulated DEGs**	**d-n-n**	0	–	–	72	0	–	–	7	0	–	–	62
		**d-d-n**	63	112	55	202	2	2	25	8	386	379	90	421
		**d-d-d**	38	17	46	37	0	–	–	2	60	99	71	140
		**n-d-d**	2	3	4	85	360	596	89	668	56	183	49	372
		**n-d-n**	88	337	76	446	177	380	86	442	60	554	68	820
		**n-n-d**	0	–	–	41	19	120	48	252	0	–	–	100
	**up-regulated DEGs**	**u-n-n**	1	5	9	55	0	–	–	14	0	–	–	47
		**u-u-n**	66	89	61	145	0	–	–	51	120	192	83	232
		**u-u-u**	26	6	60	10	0	–	–	1	71	45	75	60
		**n-u-u**	18	141	69	205	57	222	73	303	207	637	88	724
		**n-u-n**	32	247	69	357	48	300	88	341	109	832	87	961
		**n-n-u**	0	–	–	133	184	583	93	630	5	26	20	133
	**Up and down regulated DEGs**	**d-u-u**	0	–	–	3	0	–	–	0	0	–	–	3
		**u-d-d**	0	–	–	0	0	–	–	0	0	–	–	1
		**d-d-u**	0	–	–	4	0	–	–	0	0	–	–	1
		**u-u-d**	0	–	–	0	0	–	–	0	0	–	–	0
		**d-n-u**	4	5	9	53	0	–	–	3	0	–	–	16
		**u-n-d**	0	–	–	6	0	–	–	1	0	–	–	23
**Root**	**down- regulated DEGs**	**d-n-n**	82	297	69	430	0	–	–	13	140	227	75	303
		**d-d-n**	182	645	80	806	5	24	22	110	197	295	80	367
		**d-d-d**	13	9	29	31	0	–	–	4	5	23	88	26
		**n-d-d**	25	89	74	120	5	14	41	34	31	142	68	210
		**n-d-n**	34	328	71	463	65	380	78	488	36	305	75	404
		**n-n-d**	18	60	56	107	10	11	65	17	20	115	51	226
	**up-regulated DEGs**	**u-n-n**	35	262	71	367	0	–	–	7	9	14	15	93
		**u-u-n**	85	211	78	272	22	4	8	49	0	–	–	80
		**u-u-u**	13	17	22	79	17	5	42	12	23	24	38	64
		**n-u-u**	57	177	57	309	9	16	20	81	48	143	44	324
		**n-u-n**	44	262	65	406	104	309	75	413	46	116	52	224
		**n-n-u**	21	124	58	212	0	–	–	16	24	48	21	233
	**Up and down regulated DEGs**	**d-u-u**	0	–	–	3	0	–	–	0	0	–	–	3
		**u-d-d**	0	–	–	1	0	–	–	0	0	–	–	2
		**d-d-u**	0	–	–	13	0	–	–	0	9	2	40	5
		**u-u-d**	0	–	–	5	0	–	–	0	0	–	–	0
		**d-n-u**	12	64	49	130	0	–	–	0	4	4	5	87
		**u-n-d**	19	60	63	95	0	–	–	1	10	5	13	40

*^1^Expression pattern of DEGs contributing to enrichment of GO terms. d, down-regulated; u, up-regulated; n, not significantly down- or up-regulated; x-y-z, d, u, or n relating to the 35/15 (x), 35/5 (y), and 15/5 (z) sampling point comparisons.^2^GOs, number of significantly enriched GO terms.^3^DEGs contributing to GO term enrichment.^4^% total number of DEGs contributing to enriched GO terms.^5^Total number of DEGs in expression category.*

### GO Terms Associated With Leaf DEGs

Of the 137 GO terms associated with leaf transcriptomes of all three genotypes, 67 could be assigned to two main GO term hierarchies relating to chloroplast/photosynthetic metabolism, and carbohydrate/cell wall metabolism (Groups 2 and 8 in Supplementary File 4). For chloroplast/photosynthetic metabolism (25 Cellular Compartment [CC] and 9 Biological Process [BP] GO terms), the vast majority of GO terms were associated with down-regulated DEGs. Gt-9 and gt-18 started showing significant GO terms association with down-regulation during the earliest comparison (35/15), i.e., d-d-n and d-d-d, whereas for gt-17 significant association of GO terms with down-regulation was not detected until the 15/5 comparison (n-d-n and n-d-d). Thus, as a trend, down-regulation of the genes associated with the chloroplast/photosynthetic metabolism GO terms occurred earlier in gt-9 and gt-18 than in gt-17.

The second major hierarchy, consisting of carbohydrate/cell wall metabolism-related GO terms (24 BP, 8 Molecular Function [MF] and 1 CC GO terms) showed a major difference in the direction of regulation of DEGs associated with the same GO terms between genotypes. The great majority of GO terms for gt-9 and gt-18 were associated with up-regulated expression categories (mainly u-u-n and n-u-n) whilst all of the gt-17 GO terms were associated only with down-regulated expression categories (n-d-n and n-d-d), indicating major differences in the associated metabolic processes between genotypes at these point comparisons. Of the remaining GO categories, focusing on more specific terms, down-regulated categories were associated with carotenoid, cysteine and pyruvate metabolism, nitrate assimilation and response to light stimulus as well as glyoxysome and stromule CC terms. Up and down regulated GO categories were associated with alpha-amino acid, carboxylic acid and malonyl CoA biosynthetic metabolic processes. Only two GO terms were exclusively up-regulated, associated with the hexosamine pathway.

Besides the terms described above, a further 63 GO terms were not detected in all three genotypes but had highly significant *p*-values (10% most significant *p*-values within the 5% FDR) in at least one of the genotypes (Supplementary File 6). Nine of these were exclusively down-regulated in gt-9 and gt-18 and could be associated with chloroplast/photosynthetic metabolism and a further 12 were up- and down-regulated in gt-17 and gt-18 and were related to organelle compartment and organo-nitrogen/phosphate and carbohydrate metabolic processes. The remaining 42 GO terms were exclusively up-regulated, all were present in gt-17. Two were also present in gt-18 though these were not within the most significant 10% of *p*-values. These latter GO terms were associated with ribosome metabolism and location, translation and nucleotide binding. All were present in expression category n-n-u and 14 were also identified in n-u-u.

### GO Terms Associated With Root DEGs

A total of 85 GO terms were associated with all three genotypes from the root data, with the largest GO hierarchy consisting of nine GO terms (Supplementary File 5). Again, there was a noticeable difference between genotypes in terms of the expression categories in which the majority of GO terms were represented, with gt-9 and gt-18 being associated with GO terms across a number of the expression categories and gt-17 being associated with GO terms, predominantly, from only two expression categories, n-u-n and n-d-n. Thirty-seven GO terms were mostly down-regulated and fell into six main GO hierarchies with a single unconnected GO term. These were associated with peroxide activities, cellular detoxification, nitrate, phosphate and serine family amino acid metabolism and plant cell walls. Thirty-eight GO terms showed a degree of up- and down-regulation falling into 10 small GO hierarchies with five unconnected GO terms. The most specific GO descriptions were associated with membrane transport, galactose and glutamine family amino acid metabolism and metal ion binding. The remaining eight GO terms were exclusively up-regulated and fell into two small hierarchies relating to xanthine catabolism and inosine monophosphate (IMP) salvage.

A further 63 GO terms were not detected in all three genotypes but were within the 10% most significant p values within the 5% FDR (Supplementary File 7). Forty-eight of these were primarily down regulated and contained within eight GO hierarchies and two unconnected GO terms. Six of the down regulated GO hierarchies were from gt-9, in expression categories d-n-n and d-d-n and were associated with nucleotide, energy and amino acid metabolism and a single GO term with nitrate transport. A further two down-regulated hierarchies were in gt-18 and associated with the cytoskeleton and the terpenoid biosynthetic process, with an additional unconnected GO term for DNA replication. Seven primarily up-regulated GO terms in two hierarchies were in gt-9 and associated with glucan and beta-glucosidase activity and a further four GO terms in gt-17 were associated with transmembrane transport.

### KEGG Metabolic Pathways and Enzyme Codes

Differentially expressed genes associated with enzyme codes were mapped onto KEGG metabolic pathways. A total of 441 enzyme codes could be mapped to 134 pathways across all three genotypes, though with very uneven distribution (Supplementary Files 8, 9 for KEGG pathways and enzymes respectively). The five most frequently occurring pathways (excluding biosynthesis of antibiotics) were starch and sucrose metabolism, phenylpropanoid biosynthesis, amino sugar and nucleotide sugar metabolism, purine metabolism and galactose metabolism (Table 3). In terms of overall trends, DEGs contributing to starch and sucrose metabolism, galactose metabolism and glutathione metabolism were up-regulated markedly more frequently than down-regulated and DEGs contributing to phenylpropanoid metabolism, glycolysis/gluconeogenesis and cysteine and methionine metabolism showed the opposite trend. However, these overall figures can hide differences between the genotypes, particularly in relation to differences between gt-17 and gts-9/18 and DEGs from the starch and sucrose metabolism pathway; aligning with the observations from enriched GO terms, enzyme activities associated with carbohydrate and cell wall metabolism were more frequently down-regulated in leaves of gt-17 and up-regulated in gts-9/18. This can be seen specifically in terms of the most frequently occurring enzyme code associated within the starch and sucrose metabolism pathway, ec:2.4.1.12 [cellulose synthase (UDP-forming); Table 3].

**TABLE 3:** KEGG pathways and enzyme codes associated with up- and down-regulated differentially expressed genes (DEGs) responding to increasing water stress in *Brachiaria*.

		Leaf						Root						
		Up			Down			Up			Down			
		9	17	18	9	17	18	9	17	18	9	17	18	Total
**A. KEGG pathway^1^**		**Numbers of DEGs associated with pathway**												
**1**	**Starch and sucrose metabolism**	32	22	62	6	55	29	68	18	37	29	5	21	384
**2**	**Phenylpropanoid biosynthesis**	17	14	33	3	10	6	26	9	10	37	29	62	256
**3**	**Amino sugar and nucleotide sugar metabolism**	22	16	32	7	16	26	22	4	13	17	7	20	202
**4**	**Purine metabolism**	14	17	28	2	26	25	19	10	13	14	7	11	186
**5**	**Galactose metabolism**	14	9	23	5	15	17	25	17	18	9	3	9	164
**6**	**Glycolysis/Gluconeogenesis**	10	8	15	6	22	19	14	7	15	30	7	10	163
**7**	**Carbon fixation in photosynthetic organisms**	3	2	7	13	34	35	11	4	10	18	3	7	147
**8**	**Glutathione metabolism**	9	11	21	12	7	11	17	6	18	21	2	7	142
**9**	**Cysteine and methionine metabolism**	1	6	9	12	21	18	9	3	8	24	7	15	133
**10**	**Pyruvate metabolism**	9	3	12	9	27	21	9	7	13	15	3	3	131
**B. KEGG enzyme codes and activities^1^**		**Numbers of DEGs associated with enzyme activity**												
**ec:2.4.1.12**	**cellulose synthase (UDP-forming) (1)**	10	2	13	1	14	4	10	0	2	3	0	1	60
**ec:3.2.1.21**	***β*-glucosidase (1,2)**	4	2	10	0	3	2	13	4	5	1	1	5	50
**ec:1.11.1.7**	**peroxidase (3)**	7	8	16	2	4	4	10	5	4	35	27	50	172
**ec:3.2.1.14**	**chitinase (3)**	3	7	7	1	2	2	6	2	4	5	7	4	50
**ec:2.4.1.43**	**polygalacturonate 4-α-galacturonosyltransferase (4)**	2	0	5	0	2	3	4	0	2	0	0	0	18
**ec:3.6.1.3**	**Adenosinetriphosphatase (4)**	4	9	12	1	9	7	5	2	2	7	2	7	67
**ec:1.17.1.4**	**xanthine dehydrogenase (4)**	0	5	3	0	1	1	5	4	3	0	1	1	24
**ec:3.2.1.23**	***β*-galactosidase (5)**	7	1	13	1	2	1	10	3	2	0	1	2	43
**ec:2.7.1.11**	**6-phosphofructokinase (5,6)**	2	2	1	0	2	0	1	1	1	5	1	2	18
**ec:1.2.1.3**	**aldehyde dehydrogenase (NAD+) (6,10)**	1	3	4	1	2	2	1	2	5	0	1	0	22
**ec:4.1.1.31**	**phosphoenolpyruvate carboxylase (7,10)**	2	0	1	0	5	5	1	2	0	4	0	1	21
**ec:4.1.2.13**	**fructose-bisphosphate aldolase (6,7)**	0	0	2	0	2	2	0	0	0	4	0	1	11
**ec:2.5.1.18**	**glutathione transferase (8)**	7	6	11	6	5	5	12	4	10	10	1	2	79
**ec:1.1.1.44**	**phosphogluconate dehydrogenase (8)**	0	1	2	2	0	1	2	1	1	6	0	0	16
**ec:4.4.1.14**	**1-aminocyclopropane-1-carboxylate synthase (9)**	0	0	1	2	2	2	0	0	0	6	1	4	18
**ec:2.5.1.47**	**cysteine synthase (9)**	1	2	0	1	1	1	0	0	0	4	0	1	11

*A: the top 10 most frequently occurring KEGG pathways in terms of numbers of DEGs assigned to that pathway. B: the two most numerous KEGG enzyme activities associated with each of these pathways. ^1^ The numbers in brackets after the enzyme codes in B relate to KEGG pathways in A to which the enzyme code is assigned.*

The single most frequently occurring enzyme code was ec:1.11.1.7 (peroxidase; phenylpropanoid pathway), which was represented c. twice as often as the second most frequently occurring enzyme code, ec:2.5.1.18 (glutathione transferase) and showed no obvious difference between genotypes. As well as occurring most frequently, ec:1.11.1.7 was notable in that it occurred c. three times more often in the root as compared to the shoot and was down-regulated c. 2.5 times more often than it was up-regulated. Both ec:1.11.1.7 and ec:2.5.1.18-type activities would be predicted to be associated with the response to the presence of reactive oxygen species though with activities likely to be manifested in different cellular locations (apoplastic and intracellular, respectively; Dixon et al., 2009; Podgorska et al., 2017).

## Discussion

In this study, we have compared the gene expression, in response to the onset of water stress, of three Brachiaria hybrid genotypes that have previously been evaluated in terms of ability to extract water from progressively drying soil in a container-based assay. In that study gt-17 showed the greatest, gt-9 intermediate and gt-18 the least water extraction. In the experiments reported here, our focus has not been directly to suggest specific up- or down-regulated genes which have significant effects on these or other physiological responses to increasing water stress and discriminate the genotypes under study, but more to describe overall gene-expression responses, interpreted through GO and pathway analyses, in both leaves and roots. Clearly, experimental designs of this kind are comparing widely spaced ‘snapshots’ of gene expression at assay points rather than approximating continuous monitoring. Also, identified differences between tissues and genotypes do not necessarily indicate overall presences or absences of particular kinds of biological responses, just their detections at defined assay points. However, the outcomes of these studies can be valuable for indicating variability between genotypes and so point to areas which may be fruitful for further research focus in the contexts of both plant biology and, particularly in the context of this study, the exploitation of Brachiaria genetic variability for forage grass improvement.

### Drought Tolerant gt-17 Hybrid Has Distinctive Patterns of Gene Expression and GO Terms

Differentially expressed genes were identified by quantitative comparisons at three stages, 35/15, 35/5, and 15/5, representing significant and progressive changes in gene-expression profiles relating to the early stages of response to water stress. Thus, gene-models could be characterized as to their differential expression into six categories (Figure 1). From this analysis, it was apparent that overall patterns of gene expression differed both between leaves and roots and between genotypes. In particular, in the leaves, drought tolerant gt-17 tended to have more DEGs at lower EWCs relative to the genotypes with less drought tolerance, gt-9 and gt-18. For the roots, the pattern of appearance of DEGs was also distinctive for gt-17. In this latter case, the majority of DEGs were detected during the 35/5 comparison, indicating a preponderance of differential expression patterns which only reached significance over the whole analysis period, i.e., not significant within the 35/15 (early) and 15/5 (late) periods when considered separately (Figure 1).

**FIGURE 2:**
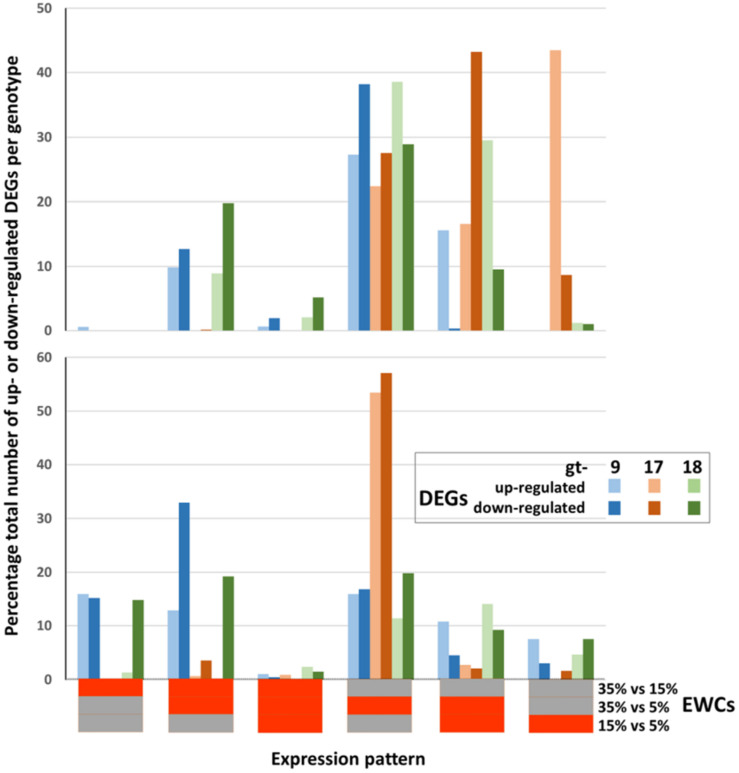
Relative proportions of differentially expressed genes (DEGs) contributing to the enrichment of GO terms across genotypes (gt-) 9, 17, and 18 and combined estimated water content (EWC) comparison points (expression patterns). The percentage proportions of up- and down-regulated DEGs identified for each individual expression pattern are indicated by the colored columns. Horizontal bars beneath the *x* axis indicate the individual EWC comparison points (indicated at the end of the *x* axis) in which the genes were differentially expressed for each expression pattern; red = differentially expressed for that EWC comparison point; gray = not differentially expressed for that EWC comparison point. E.g., the first expression pattern indicates the percentage proportion of the total number of up- and down-regulated DEGs for each genotype that were differentially expressed for the 35% vs. 15% comparison point (red) but were not differentially expressed for the 35% vs. 5% and 15% vs. 5% comparison points (gray).

The overall trends seen in Figure 1 can be compared with those generated when just the DEGs which contribute to the enrichment of GO terms are included (c. 70% of the total DEGs for leaves and c. 62% of the total DEGs for roots; Figure 2). The trends are very similar when considering all DEGs and only those DEGS which contribute to the significant enrichment of GO terms. Thus, the DEGs at the different comparison stages are contributing proportionally to biological processes as indicated through GO terms. However, while the relative proportions across comparison periods were consistent, there was some variation in terms of genotype as indicated in Table 4. Particularly, (a) proportionally fewer of the total number of up- and down-regulated leaf DEGs in gt-9 contribute to the enrichment of GO terms compared to gt-17 and gt-18 and (b) for gt-18, only 34% of the up-regulated root DEGs contribute to GO term enrichment whereas the equivalent figure for down-regulated DEGs was 72%. The equivalent comparisons for gt-9 and gt-17 were far more even.

**TABLE 4:** The relative proportions of differentially expressed genes (DEGs) which contributed to the significant enrichment of GO terms according to genotype, organ and direction of regulation.

		DEGs					
		Up-regulated		Down-regulated		Up-/down-regulated	
Organ	Genotype	Total	% associated with GOs	Total	% associated with GOs	Total	% associated with GOs
**Leaves**	**9**	905	54	883	53	1788	54
	**17**	1340	82	1379	80	2719	81
	**18**	2157	80	1915	63	4072	72

**Roots**	**9**	1645	64	1957	73	3602	69
	**17**	578	58	666	64	1244	61
	**18**	1018	34	1536	72	2554	57

**FIGURE 3:**
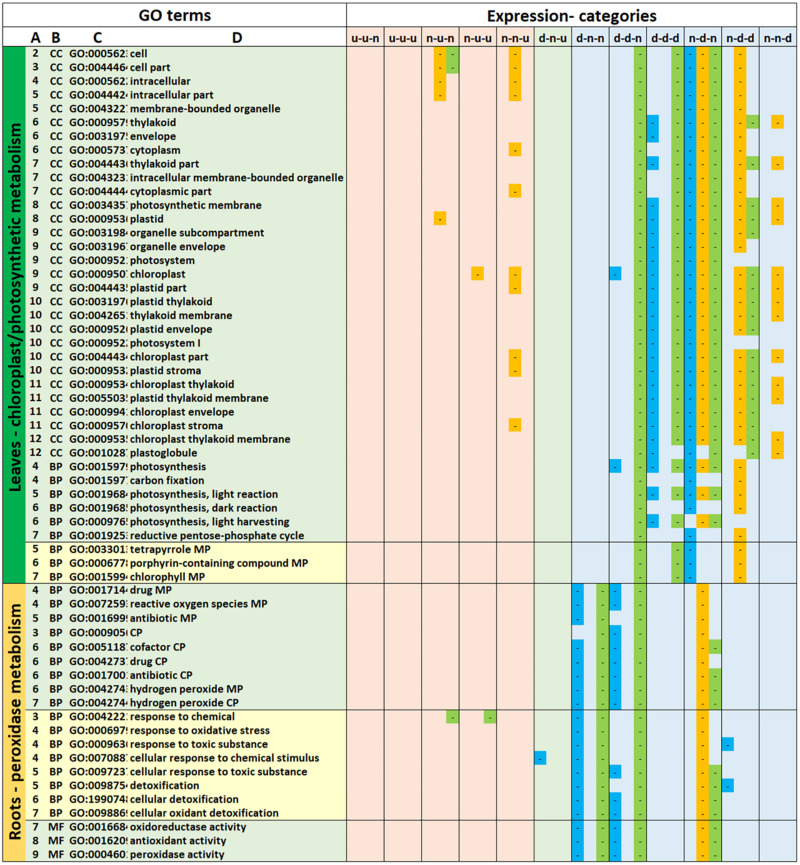
GO terms identified in all three genotypes relating to photosynthetic metabolism in the leaves and peroxide and cellular detoxification processes in the root. GO Terms **(A)** relative positions of GO terms linked through hierarchies visualized at *https://www.ebi.ac.uk/QuickGO/slimming*. **(B)** GO category, BP, biological process; CC, cell compartment; MF, molecular function. **(C)** GO description (^1^MP, metabolic process; ^2^CP, catabolic process). Expression Categories: Expression patterns of differentially expressed genes contributing to enrichment of GO terms. d, down-regulated; u, up-regulated; n, not significantly down- or up-regulated; x-y-z, d, u, or n relating to the 35/15 (x), 35/5 (y), and 15/5 (z) sampling point comparisons. The first column for each expression category indicates whether a GO term(s) was significantly enriched for gt-9 (blue), the second column for gt-17 (orange) and the third for gt-18 (green).

Overall differences in differential gene expression itself and the contribution of DEGs to GO terms indicate that there can be substantial variation between genotypes and tissues. This genotype-dependent manifestation of the variation in the overall patterns of DEGs and GO terms can be seen when looking at particular biological examples, as illustrated in Figure 3. The data shown focuses on the GO terms predominantly associated with down-regulation relating to chloroplast/photosynthetic metabolism in the leaves and peroxidase metabolic processes and cellular detoxification in the roots. While the responses are not identical in leaves and roots, the genotype-specific trend is the same with the down-regulation of the indicated biological process commencing (i.e., reaching significance in terms of enrichment) earlier for gt-9 and gt-18 than for gt-17. In fact, when all GO terms are considered (Supplementary Files 2, 3) a total of 1611 enriched GO terms are associated with DEGs up- or down-regulated in the 35/15 comparison period for gts-9 and gt-18 but only 46 for gt-17.

**FIGURE 4:**
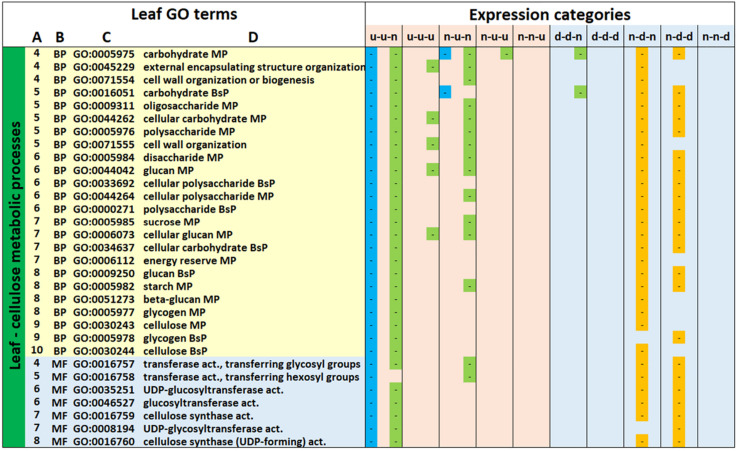
GO terms identified in all three genotypes relating to carbohydrate and cell wall metabolism in the leaf. Leaf GO Terms **(A)** relative positions of GO terms linked through hierarchies visualized at *https://www.ebi.ac.uk/QuickGO/slimming*. **(B)** GO category, BP, biological process; CC, cell compartment; MF, molecular function. **(C)** GO description (MP, metabolic process; CP, catabolic process; BsP, biosynthetic process; act., activity). Expression Categories: Expression patterns of differentially expressed genes contributing to enrichment of GO terms. d, down-regulated; u, up-regulated; n, not significantly down- or up-regulated; x-y-z, d, u or n relating to the 35/15 (x), 35/5 (y), and 15/5 (z) sampling point comparisons. The first column for each expression category indicates whether a GO term(s) was significantly enriched for gt-9 (blue), the second column for gt-17 (orange) and the third for gt-18 (green).

An additional example of the distinct responses of gt-17 is indicated by the leaf GO hierarchies for which the terminal GO terms were GO:0030244 (cellulose biosynthetic process) and GO:0016760 (cellulose synthase [UDP-forming]) (Figure 4). These were predominantly associated with up-regulation at the first comparison point (35/15) for gt-9 and gt-18 and down-regulation for gt-17 over the 35/5 comparison point; for most of these gt-17 GO terms, there was association with further down-regulation during the 15/5 comparison point. The GO terms associated with up-regulation in this hierarchy for gt-9 (69 DEGs) and gt-18 (217 DEGs) were enriched by a total of 234 individual gene models, 52 of which were common to both genotypes (representing 75% of the total for gt-9). The down-regulated gene models for gt-17 for this hierarchy contained 154 gene models, 23 of which were also in the up-regulated DEGs for gt-9 and gt-17. Thus, the DEGs being differentially regulated in opposite directions in gts-9/18 and in gt-17 had some overlap but were, in the main, different subsets of genes relating to the same GO terms.

**FIGURE 5:**
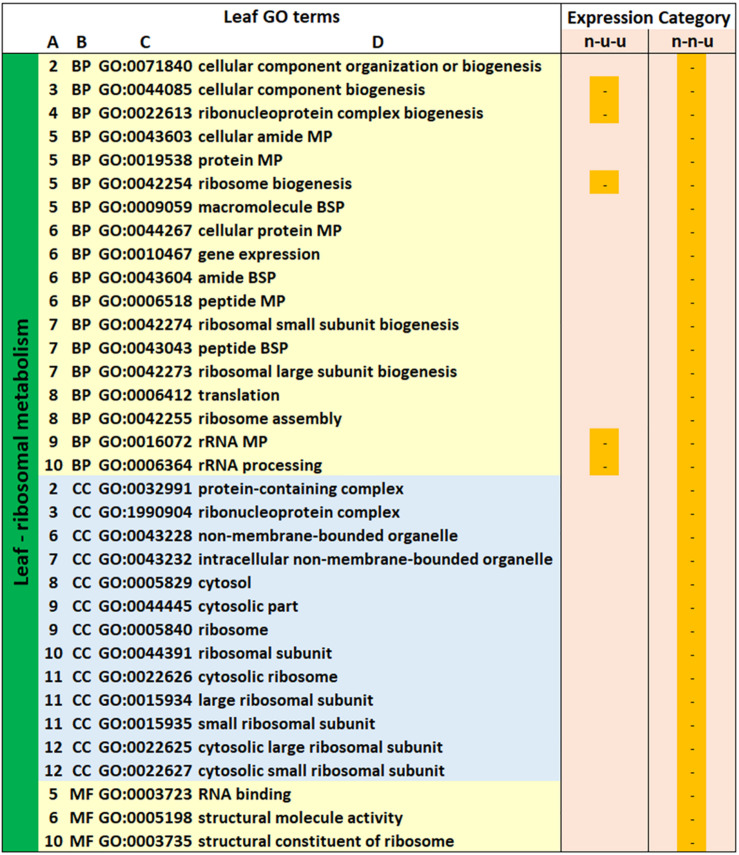
GO terms identified relating to ribosomal metabolism and location in the leaf. Leaf GO Terms **(A)** relative positions of GO terms linked through hierarchies visualized at *https://www.ebi.ac.uk/QuickGO/slimming.* **(B)** GO category, BP, biological process; CC, cell compartment; MF, molecular function. **(C)** GO description (MP, metabolic process; BsP, biosynthetic process). Expression Categories: Expression patterns of differentially expressed genes contributing to enrichment of GO terms. d, down-regulated; u, up-regulated; n, not significantly down- or up-regulated; x-y-z, d, u, or n relating to the 35/15 (x), 35/5 (y), and 15/5 (z) sampling point comparisons. The first column for each expression category indicates whether a GO term(s) was significantly enriched for gt-9 (blue), the second column for gt-17 (orange) and the third for gt-18 (green). Only gt-17 had significantly enriched GO terms.

KEGG pathway analysis of these sets of up- (gts-9/18) and down- (gt-17) regulated gene models mapped onto 16 and 19 enzyme codes, respectively, in the Starch and Sucrose Metabolism pathway including 13 gene models in both of gts-9/18 and gt-17 assigned to ec:2.4.1.12 (cellulose synthase [UDP-forming]) but regulated in the opposite direction (Supplementary Figure 6). Additionally, the down-regulated gt-17 gene model set contained nine annotations describing ‘starch synthase’ and four describing ‘sucrose-phosphate synthase’, indicating down-regulation of key enzymes of energy metabolism, additional to those more directly involved in cellulose synthesis. Thus, while in gt-17 GO terms relating to photosynthesis and carbohydrate metabolism are all associated with down-regulation during the same comparison points, for gt-9 and gt-18, the association with down-regulation of photosynthesis-related GO terms is accompanied by association with up-regulation of the cell-wall and carbohydrate-related GO terms (Figures 3, 4). Again, this suggests underlying differences between gts-9/18 and gt-17 in their metabolic responses to the progression of water limitation. Similarly, one particular hierarchy of GO terms, relating to ribosomal metabolism, was present for gt-17 but completely absent for gts-9/18 (Figure 5) with significant enrichment for all GO terms in the 15/5 comparison period and for five of the terms also in the 35/5 comparison period. A total of 313 up-regulated gene models contributed to the enrichment of these GO terms, of which 82 were annotated as ribosomal proteins. There was also up-regulation of 15 ribosomal protein genes from gt-18 in the u-n-n, u-n-u and u-u-n expression categories, though this was not sufficient for significant enrichment of the related GO terms. Only the ribosomal protein genes were DEGs from gt-9 and all were down-regulated. The basic function of ribosomal proteins is protein synthesis as part of the ribosomal complex. However, the potential role of ribosomal proteins in conferring abiotic stress, and particularly drought tolerance, has been reported (Moin et al., 2017). Thus, this represents another interesting contrast between gt-17 and gts-9/18.

As referred to earlier, it has been observed that gt-17 can be more drought tolerant than gt-9 or gt-18 in container-based trials conducted as a part of the CIAT breeding program. Clearly, extrapolating from the present growth-room/artificial-media based experiment to a container-based trial, let alone to a field situation has to be done with extreme caution. However, the results reported here do indicate that genetic variation between the three genotypes has significant and measurable impacts on patterns of gene expression in response to water stress. The fact that such variation should exist is not surprising and there are numerous examples of gene-expression studies and QTL analyses for different aspects of drought tolerance in forage and other grasses (e.g., Alm et al., 2011; Merewitz et al., 2014; Baldoni et al., 2016; Jiang et al., 2017; Liang et al., 2017; Zhu et al., 2017; Cheruiyot et al., 2018; Hu et al., 2018; Ji et al., 2018; Kumar et al., 2018; Ling et al., 2018; Ma et al., 2018; Chaichi et al., 2019; Dinkins et al., 2019; Fradera-Sola et al., 2019). However, in the context of Brachiaria breeding, these genotypes were selected from a cultivar development program and the results suggest selectable variation with measurable impacts.

### EC:1.11.1.7 – Peroxidase (Phenylpropanoid Biosynthesis Pathway) Down-Regulated Genes Are Over-Represented in the Root in All Genotypes

One interesting observation which was not genotype specific, was that gene models which could be assigned to ec:1.11.1.7 (peroxidase) were strongly over-represented in down-regulated DEGs in the root, in comparison to other enzyme classes as a whole (Table 3 and Supplementary File 9) and other enzyme classes within the phenylpropanoid biosynthesis pathway (Table 5 and Supplementary Figure 7). In a similar study (Fradera-Sola et al., 2019) focusing on the C_3_ forage grass perennial ryegrass (*Lolium perenne*), the same enzyme class was also over-represented in down-regulated expression categories in root tissue and, to a lesser extent, in the leaves. Peroxidase genes and activities (though not necessarily ec:1.11.1.7) have been reported as being differentially expressed in a number of studies (Csiszár et al., 2012; Ranjan et al., 2012; Hu et al., 2018; Chaichi et al., 2019) pertaining to drought stress, in some cases suggesting that relatively higher levels of peroxide transcripts are detected in more drought tolerant genotypes under drought stress (Merewitz et al., 2014). Peroxidases play pivotal roles in both cytosolic and apoplastic responses to reactive oxygen species and, in the apoplast, specifically lignification and elasticity of secondary cell walls (reviewed in Podgorska et al., 2017; Meents et al., 2018). In the present study it is particularly the down-regulation of apoplastic peroxidases that is implicated which might suggest weakening of the mechanical properties of the root cell wall as water stress increases. This may allow newly divided cells to expand more readily under water-limited conditions (Tenhaken, 2015), but may also reflect that root cell walls more generally (rather than just at the expansion zone at the root tip) may develop different mechanical properties in response to the drying of the medium in which they are growing.

**TABLE 5:** The number of differentially expressed genes (DEGs) associated with enzyme codes from the KEGG Phenylpropanoid Biosynthesis pathway according to genotype, organ and direction of regulation.

			KEGG enzyme activity and code					
Tissue	Genotype	direction of DEG regulation	ec:1.1.1.195 cinnamyl-alcohol dehydrogenase	ec:3.2.1.21 β-glucosidase	ec:6.2.1.12 coumarate-CoA ligase	ec:1.11.1.7 peroxidase	ec:1.2.1.44 cinnamoyl-CoA reductase	ec:2.1.1.68 caffeate O-methyltransferase
**leaf**	**gt-9**	**up**	3	4	3	7	–	–
		**down**	–	–	–	2	1	–
	**gt-17**	**up**	3	2	1	8	–	–
		**down**	2	3	1	4	–	–
	**gt-18**	**up**	5	10	2	16	–	–
		**down**	–	2	–	4	–	–
	**All gt**	**up**	11	16	6	31	–	–
		**down**	2	5	1	10	1	–
**root**	**gt-9**	**up**	2	13	–	10	1	–
		**down**	–	1	1	35	–	–
	**gt-17**	**up**	–	–	4	5	–	–
		**down**	1	1	–	27	–	–
	**gt-18**	**up**	1	5	–	4	–	–
		**down**	3	5	1	50	1	2
	**All gt**	**up**	3	18	4	19	1	–
		**down**	4	7	2	112	1	2

## Conclusion

We have undertaken a study comparing differential gene-expression, in relation to the onset of water stress, across three Brachiaria hybrid genotypes with contrasting physiological responses to water deficit and identified a range of GO terms linked to biological responses in leaves and roots. In leaves, GO term hierarchies relating to photosynthetic metabolism and carbohydrate metabolism were well supported as were antioxidant and peroxidase activities in roots. The two most striking aspects of the results were, (a) both overall proportions of DEGs and specific related GO terms enriched in all three genotypes varied in a genotype-dependent manner in relation to expression categories, with gt-9 (intermediate water stress tolerance) and gt-18 (susceptible to water stress) being more similar to each other than gt-17 (tolerant of water stress), and (b) the GO terms relating to carbohydrate and cell wall metabolism in the leaves were enriched by up-regulated DEGs for gt-9 and gt-18, but down-regulated DEGs for gt-17 at the EWC points assayed. Additionally, across all genotypes, analysis of enzyme activities relating to DEGs using the KEGG database, indicated a large excess of down-, as compared to up-regulated DEGs with likely apoplastic peroxidase activities in the roots as water stress increased. This suggests that changes in root cell-wall architecture may be an important component of the response to water stress in these genotypes. In taking this work forward, it will be particularly interesting to see if overall time-course patterns of gene expression can be correlated with response to water stress in a wider range of Brachiaria genotypes. If so, it may be possible to identify the genetic basis by which the syndrome of metabolic events, which manifest with increasing water stress are initiated and enacted.

## Data Availability Statement

The data presented in this study can be found in the European Nucleotide Archive repository under the study accession number PRJEB41722 (https://www.ebi.ac.uk/ena/browser/view/PRJEB41722). Additional data that support the results are included within the article and its additional files. Other relevant materials are available from the corresponding authors on reasonable request. Plant material is publicly available at the CIAT Genebank (genebanks.org/resources/crops/forages-grass/).

## Author Contributions

JAC, JC, MW, and JA conducted the initial experiments to identify breeding selections with good, intermediate, and poor tolerance to drought to be used in this study. NF-F and IA conceived and designed the experiments and computational analyses. CJ, AT, DG, JH, YF, and NF-F performed the experiments. JDV, MW, JD, MB, FC, JA, JAC, and JC contributed expertise and reagents. CJ, YF, AT, IA, and NF-F analyzed the data. CJ, NF-F, and IA drafted the initial version. NF-F and IA finalized the drafting with help from all authors. All the authors have read and approved this manuscript.

## Conflict of Interest

The authors declare that the research was conducted in the absence of any commercial or financial relationships that could be construed as a potential conflict of interest.

## Footnotes

1 https://ciat.cgiar.org/what-we-do/forages-livestock/

2 https://www.genebanks.org/resources/crops/forages-grass/
