# Supplementary material for: A Comparison of Differential Gene Expression in Response to the Onset of Water Stress Between Three Hybrid Brachiaria Genotypes
Source: Front Plant Sci. 2021 Mar 19;12:637956. doi: 10.3389/fpls.2021.637956 (PMC8017340; doi:10.3389/fpls.2021.637956)
Supplement: Supplementary file 10 [file Data_Sheet_1.docx]

Supplementary Material

# Supplementary Data

- Supplementary file 1: suppl_file1.xlsx. The complete list of DEGs in leaves and roots according to expression category and genotype. Genes annotation obtained from {Worthington, 2020 #152}.
- Supplementary file 2: suppl_file2.xlsx. The complete list of enriched GO terms for each genotype at each expression category using a 5% FDR in leaf tissue.
- Supplementary file 3: suppl_file3.xlsx. The complete list of enriched GO terms for each genotype at each expression category using a 5% FDR in root tissue.
- Supplementary file 4: suppl_file4.xlsx. The list of GO terms identified for all 3 genotypes at any expression category using a 5% FDR in leaf tissue.
- Supplementary file 5: suppl_file5.xlsx. The list of GO terms identified for all 3 genotypes at any expression category using a 5% FDR in root tissue.
- Supplementary file 6: suppl_file6.xlsx. GO terms identified in any genotype with -log_10_ P ≥ 10.4 in leaf tissue.
- Supplementary file 7: suppl_file7.xlsx. GO terms identified in any genotype with -log_10_ P ≥ 10.4 in root tissue.
- Supplementary file 8: suppl_file8.xlsx. The number of DEGs associated with individual KEGG pathways according to genotype, organ and direction of regulation.

.

- Supplementary file 9: suppl_file9.xlsx. The number of DEGs associated with individual KEGG enzymes according to genotype, organ and direction of regulation.

# Supplementary Figures and Tables

## Supplementary Figures

##
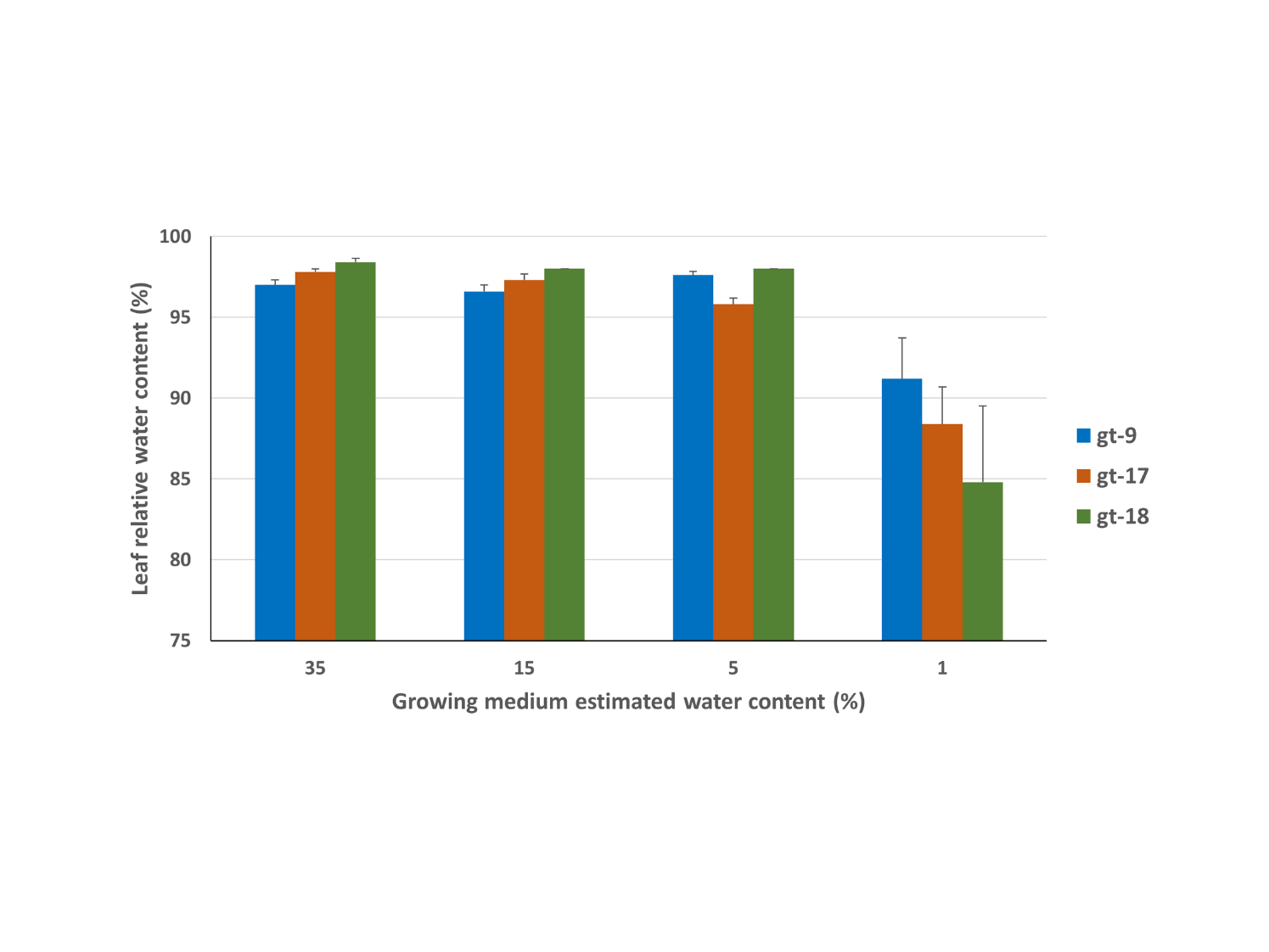
Supplementary Figure 1. Relative leaf water content for Brachiaria hybrid genotypes gt-9, gt-17 and gt-18 as estimated water content of the growing medium decreased.

## .

**Supplementary Figure 2.** The DESeq2 rlog transformation distribution of counts for each of the 3 replicates and sampling point. A, B, C are from leaf transcriptome on gt-9, gt-17 and gt-19 respectively; and D, E F are from root transcriptomes on on gt-9, gt-17 and gt-19 respectively.


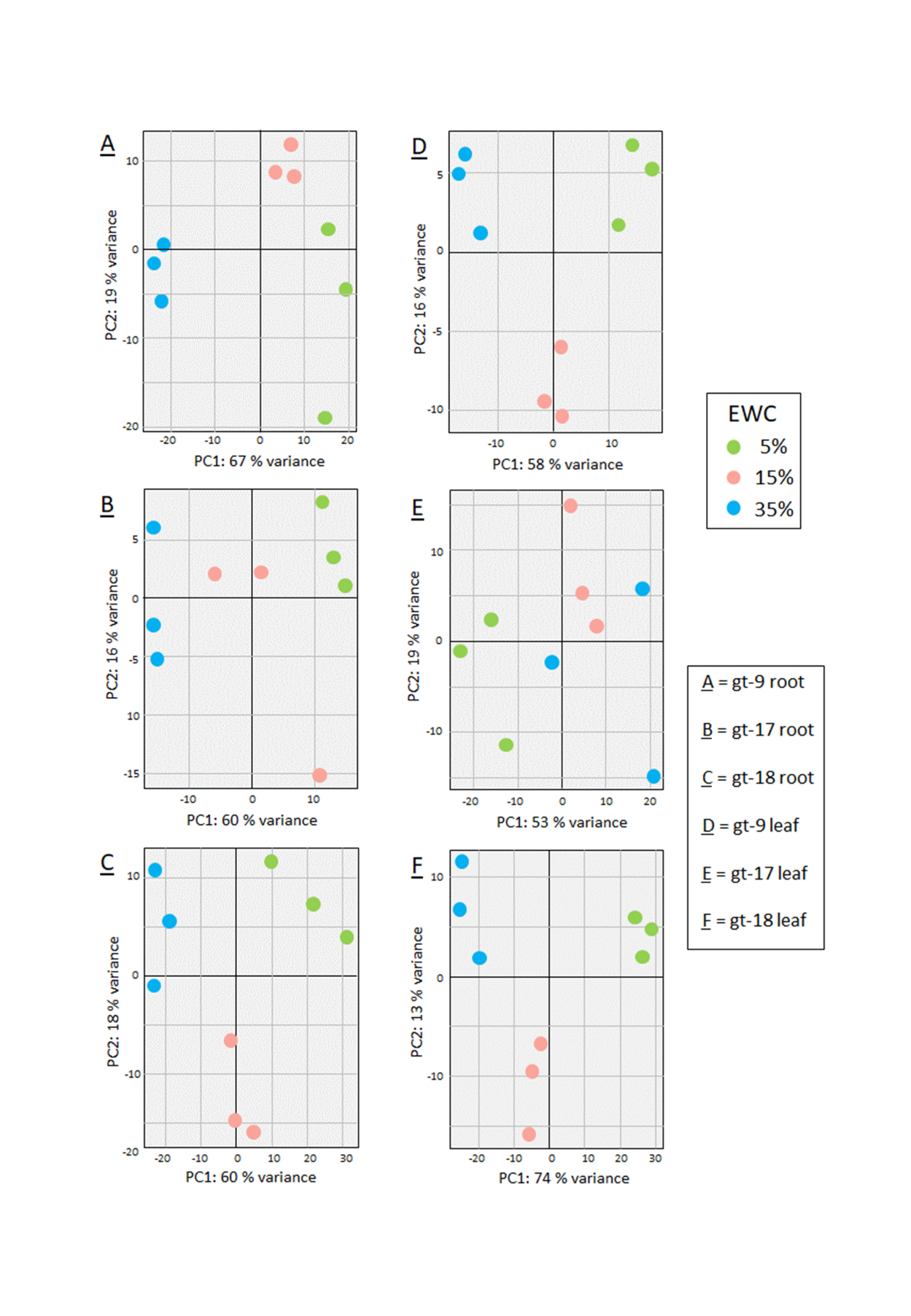


**Supplementary Figure 3.** Principal component analysis illustrating variability among replicates from leaf and root transcriptomes for the three genotypes.


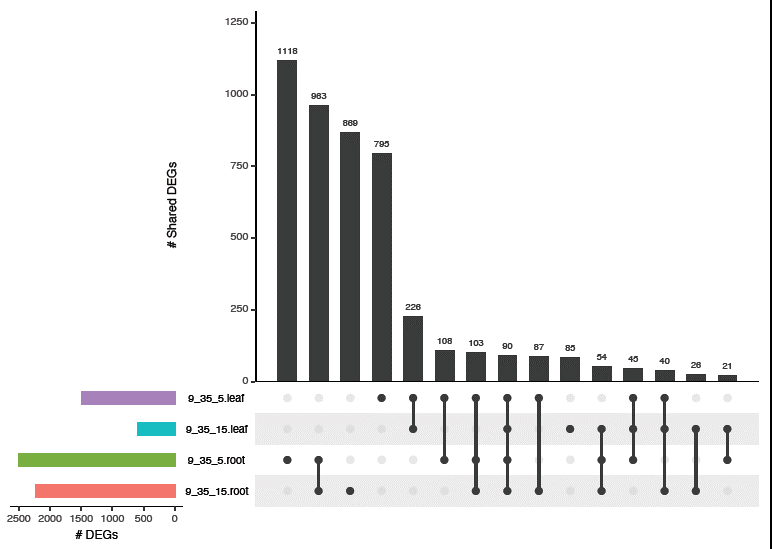


**Supplementary Figure 4**. The number of shared DEGs across sampling comparisons and tissues for gt-9. This illustrates the general trend that only a limited number of genes were differentially expressed in both leaves and roots.


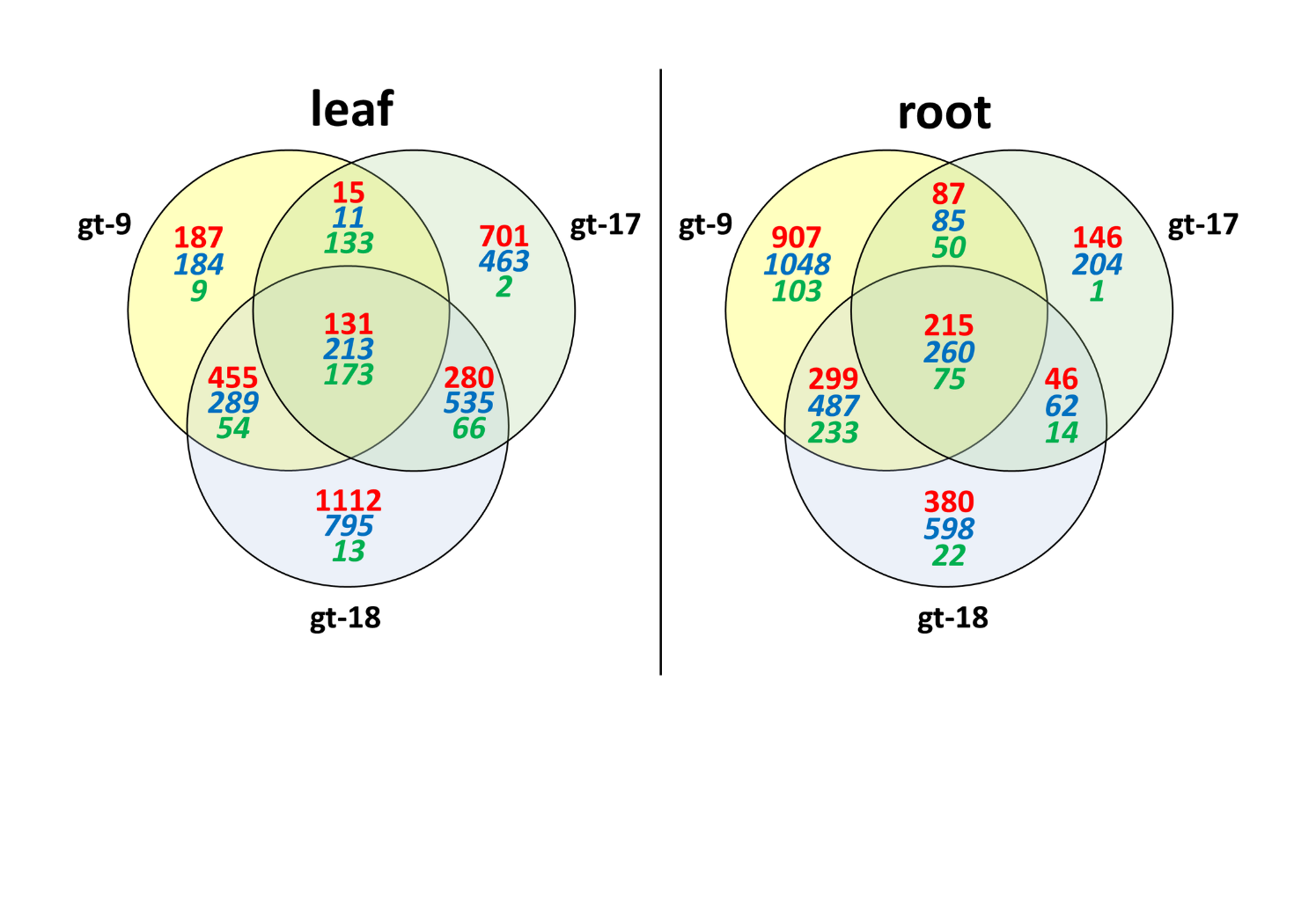


**Supplementary Figure 5**. The number of up-regulated (red), down-regulated (blue) and up- and down-regulated (green) differentially expressed genes common across or exclusive to genotypes (gt-) 9, 17 and 18 across all of the comparison stages.

**
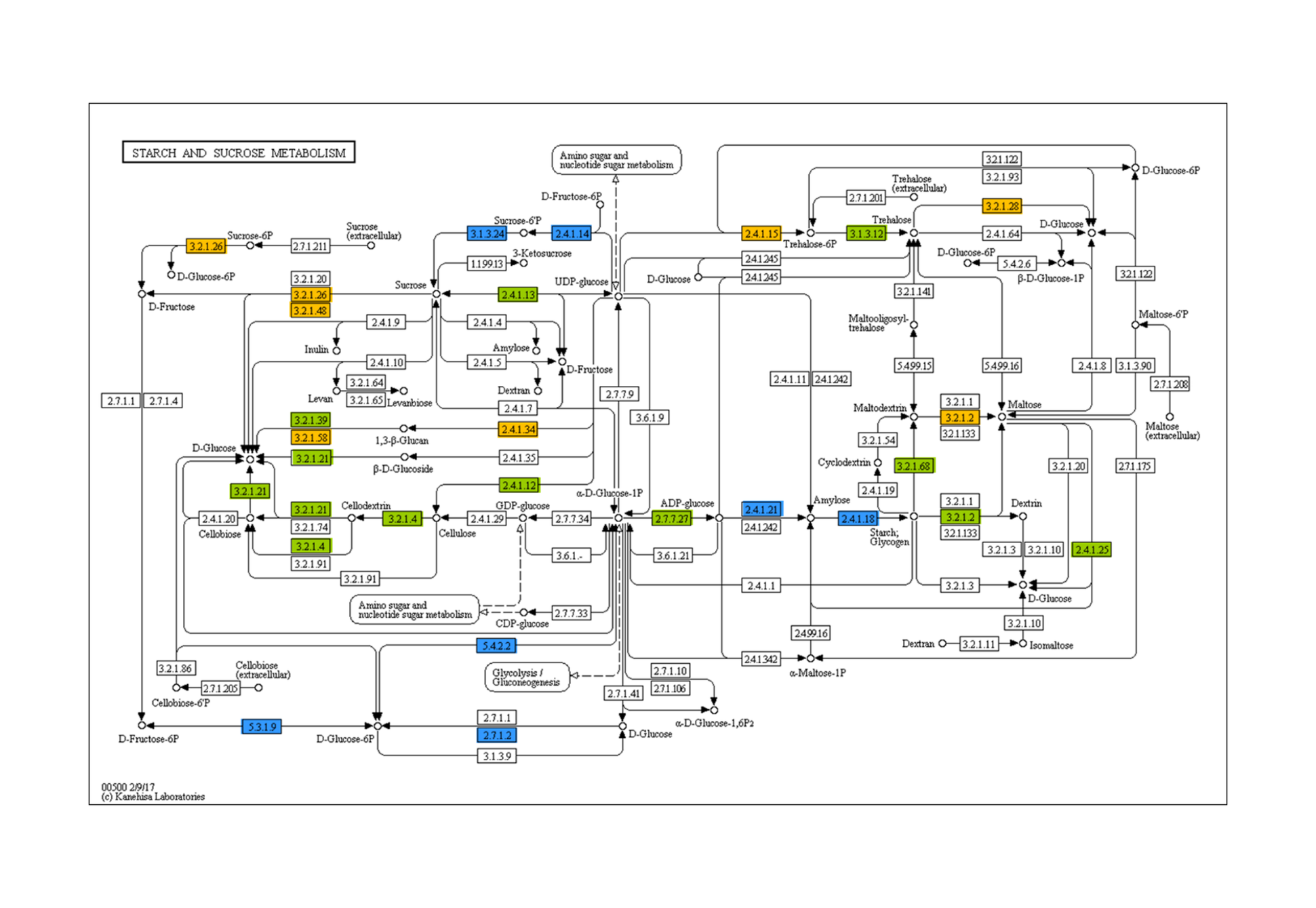
**

**Supplementary Figure 6.** Enzymes activities in the Starch and Sucrose Metabolism Pathway (obtained from <https://www.genome.jp/kegg-bin/show_pathway?map00500>) regulated in different directions in gts-9/18 and gt-17. Colour coded enzyme activities were up- (orange), down- (blue) or up- and down- (green) regulated according to enzyme activities assigned to DEGs detected in this study.


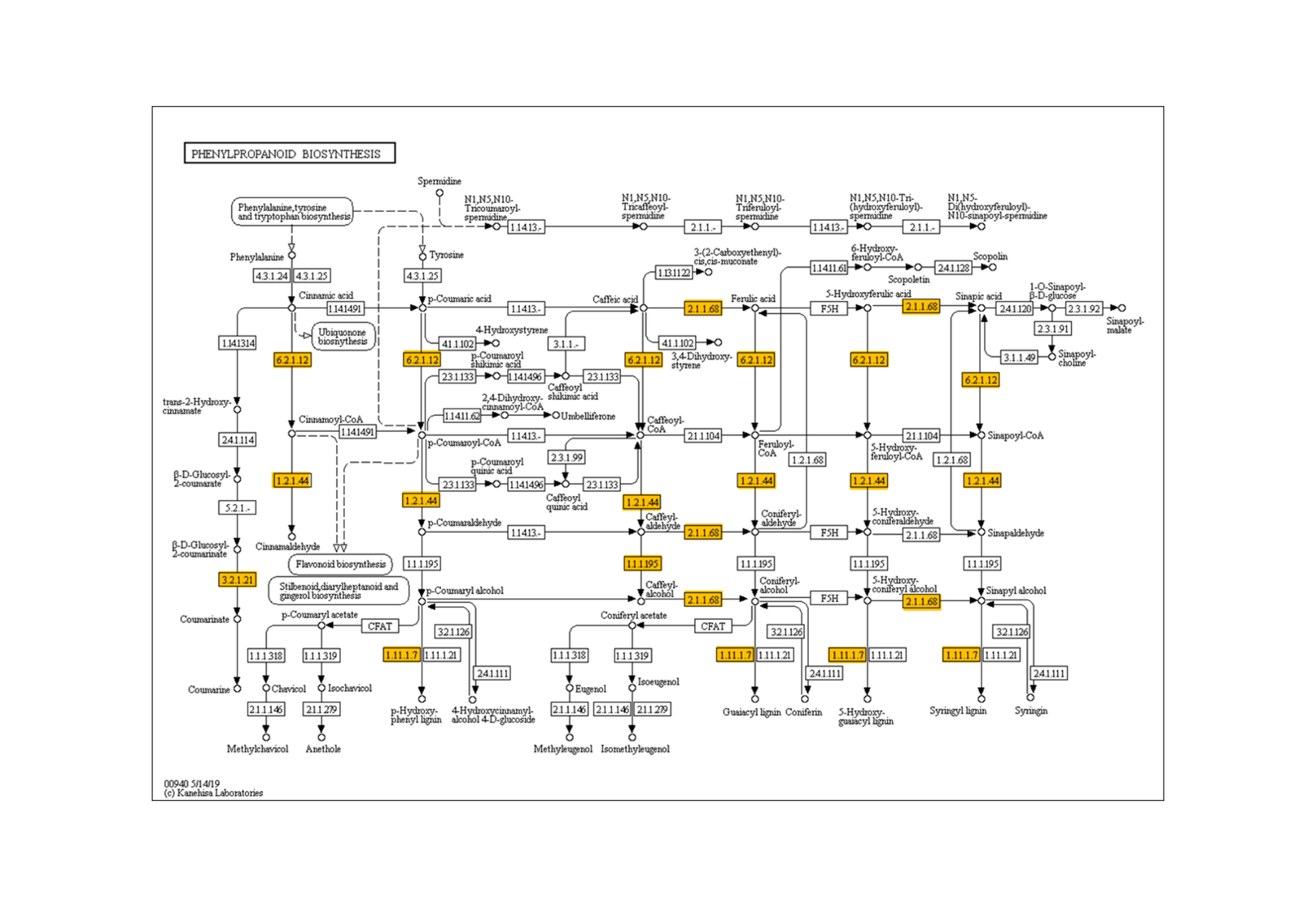


**Supplementary Figure 7.** Enzymes activities in the Phenylpropanoid Biosynthesis Pathway (obtained from <https://www.genome.jp/kegg-bin/show_pathway?map00940>). Colour coded enzyme activities were assigned to DEGs detected in this study.

## Supplementary Tables

| **Supplementary Table 1.**  Brachiaria accessions used for the generation and comparison of transcriptomes and differentially expressed genes associated with increasing water-stress. | | |
| --- | --- | --- |
| **Accession** | **Abbreviation** | **Drought tolerance^1^** |
| Br12/3659-17 | gt-17 | Good |
| Br12/2360-9 | gt-9 | Intermediate |
| Br12/3868-18 | gt-18 | Poor |
|  | | |
| ^1^Drought tolerance indicates the relative performance of the genotypes in the presence of drought conditions when tested during the CIAT evaluation programme. | | |

| **Supplementary Table 2. A.** The average numbers of total and processed paired sequencing reads across replicates according to genotype and sampling point (estimated growing medium water content; EWC). **B**. Range of % alignments of processed reads to the reference genome across replicates according to genotype and sampling point | | | | | | |
| --- | --- | --- | --- | --- | --- | --- |
| **A** | **Average numbers of reads** | | | | | |
|  | **35 % EWC** | | **15 % EWC** | | **5 % EWC** | |
| **Leaf** | **Total** | **Processed** | **Total** | **Processed** | **Total** | **Processed** |
| **gt-17** | 13207351 | 13005003 | 11295245 | 11116518 | 11570161 | 11389779 |
| **gt-9** | 11267973 | 11105520 | 11140108 | 10951529 | 11125445 | 10938498 |
| **gt-18** | 10821598 | 10625209 | 11246647 | 11039917 | 12022034 | 11831204 |
|  |  | |  | |  | |
| **Roots** |  | |  | |  | |
| **gt-17** | 11896249 | 11728093 | 10847917 | 10672392 | 11042582 | 10853348 |
| **gt-9** | 10252555 | 10079815 | 10915442 | 10731902 | 10799173 | 10596343 |
| **gt-18** | 11942424 | 11728093 | 12438691 | 12202566 | 11422363 | 11248249 |
|  |  | |  | |  | |
| **B** | **Alignment rate (%)** | | | | | |
| **Leaf** |  | |  | |  | |
| **gt-17** | 74 - 64 | | 73 - 72 | | 74 - 63 | |
| **gt-9** | 75 - 64 | | 74 - 69 | | 70 - 58 | |
| **gt-18** | 76 - 69 | | 72 - 62 | | 74 - 72 | |
|  |  | |  | |  | |
| **Roots** |  | |  | |  | |
| **gt-17** | 47 - 44 | | 61 - 45 | | 61 - 45 | |
| **gt-9** | 70 - 64 | | 63 - 61 | | 60 - 48 | |
| **gt-18** | 72 - 63 | | 65 - 63 | | 70 - 61 | |

| **Supplementary Table 3.** The completeness of the different transcriptomes (%) as estimated through the BUSCO core gene set. Data is represented in terms of the % of complete, partial and missing BUSCO core genes according to genotype, tissue and sampling point (estimated growing medium water content; EWC). The completeness of the Brachiaria reference genome is given for comparison. | | | | | |
| --- | --- | --- | --- | --- | --- |
|  |  | | **BUSCO core gene set (%)** | | |
| **Genotype** | **Tissue** | **EWC** | **Complete** | **Partial** | **Missing** |
| **gt-17** | **Leaf** | **35** | 55.7 | 20.0 | 24.3 |
|  |  | **15** | 47.8 | 18.7 | 33.5 |
|  |  | **5** | 57.9 | 18.6 | 23.5 |
|  |  |  |  |  |  |
|  | **Root** | **35** | 48.1 | 19.5 | 32.4 |
|  |  | **15** | 58.3 | 18.6 | 23.1 |
|  |  | **5** | 57.1 | 18.9 | 24.0 |
|  |  |  |  |  |  |
| **gt-9** | **Leaf** | **35** | 59.2 | 20.2 | 20.6 |
|  |  | **15** | 59.0 | 20.0 | 21.0 |
|  |  | **5** | 57.9 | 21.6 | 20.5 |
|  |  |  |  |  |  |
|  | **Root** | **35** | 60.1 | 19.8 | 20.1 |
|  |  | **15** | 58.9 | 18.8 | 22.2 |
|  |  | **5** | 56.0 | 21.1 | 22.9 |
|  |  |  |  |  |  |
| **gt-18** | **Leaf** | **35** | 51.4 | 18.8 | 29.8 |
|  |  | **15** | 60.1 | 19.6 | 20.3 |
|  |  | **5** | 65.9 | 19.1 | 15.0 |
|  |  |  |  |  |  |
|  | **Root** | **35** | 64.9 | 19.3 | 15.8 |
|  |  | **15** | 63.5 | 17.9 | 18.6 |
|  |  | **5** | 61.6 | 18.1 | 20.3 |
| **Reference genome** | | | 68.6 | 15.9 | 15.5 |
